# De novo assembly of a fruit transcriptome set identifies AmMYB10 as a key regulator of anthocyanin biosynthesis in *Aronia melanocarpa*

**DOI:** 10.1186/s12870-022-03518-8

**Published:** 2022-03-25

**Authors:** Jonathan D. Mahoney, Sining Wang, Liam A. Iorio, Jill L. Wegrzyn, Matthew Dorris, Derek Martin, Bradley W. Bolling, Mark H. Brand, Huanzhong Wang

**Affiliations:** 1 Department of Plant Science and Landscape Architecture, University of Connecticut, Storrs, CT 06269 USA; 2 Department of Ecology and Evolutionary Biology, University of Connecticut, Storrs, CT 06269 USA; 3 Institute for Systems Genomics, University of Connecticut, Storrs, CT 06269 USA; 4 Department of Food Science, University of Wisconsin, Madison, WI 53706 USA

**Keywords:** Rosaceae, Aronia, Anthocyanin, Transcriptome, Transcription factor

## Abstract

*Aronia* is a group of deciduous fruiting shrubs, of the Rosaceae family, native to eastern North America. Interest in *Aronia* has increased because of the high levels of dietary antioxidants in *Aronia* fruits. Using Illumina RNA-seq transcriptome analysis, this study investigates the molecular mechanisms of polyphenol biosynthesis during *Aronia* fruit development. Six *A*. *melanocarpa* (diploid) accessions were collected at four fruit developmental stages. De novo assembly was performed with 341 million clean reads from 24 samples and assembled into 90,008 transcripts with an average length of 801 bp. The transcriptome had 96.1% complete according to Benchmarking Universal Single-Copy Orthologs (BUSCOs). The differentially expressed genes (DEGs) were identified in flavonoid biosynthetic and metabolic processes, pigment biosynthesis, carbohydrate metabolic processes, and polysaccharide metabolic processes based on significant Gene Ontology (GO) biological terms. The expression of ten anthocyanin biosynthetic genes showed significant up-regulation during fruit development according to the transcriptomic data, which was further confirmed using qRT-PCR expression analyses. Additionally, transcription factor genes were identified among the DEGs. Using a transient expression assay, we confirmed that *AmMYB10* induces anthocyanin biosynthesis. The de novo transcriptome data provides a valuable resource for the understanding the molecular mechanisms of fruit anthocyanin biosynthesis in *Aronia* and species of the *Rosaceae* family.

**Supplementary Information:**

The online version contains supplementary material available at 10.1186/s12870-022-03518-8.

## Introduction

Plants in the *Rosaceae* family include a number of economically important fruit crops beneficial for human nutrition [1]. Common pome fruits include *Malus* Mill. (apple), *Pyrus* L. (pear) and *Cydonia* Mill. (quince), along with less commonly known fruits including *Sorbus* L. (mountain ash), *Aronia* Med. (chokeberry)*, Amelanchier* Med. (serviceberry)*, Crataegus* L. (hawthorn), and several other woody plants [2]. The three commonly accepted *Aronia* species include *A. arbutifolia* (L.) Pers., red chokeberry (tetraploid); *A. melanocarpa* (Michx.) Elliott, black chokeberry (diploid and tetraploid); and *A. prunifolia* (Marshall) Reheder, purple chokeberry (triploid and tetraploid). A fourth species of *Aronia* has been recognized as *A. mitschurinii* (A.K. Skvortsov & Maitul) (tetraploid) and it is used in commercial fruit production, usually as the cultivars ‘Viking’ or ‘Nero’ [3]. Diploid *A. melanocarpa* reproduce sexually and are most commonly found in the Northeastern United States, primarily in the New England region [4, 5]. These fruits can easily be identified by the presence of dark-colored fruits in mid- to late summer.

Interest in *Aronia* is high because of the high levels of polyphenolic compounds, primarily anthocyanins, and high antioxidant activity [6–8]. A diet rich in flavonoid and polyphenolic compounds is thought to play an essential role in preventing diseases [9]. *Aronia* fruits have been shown to have numerous health-promoting activities such as antioxidant, cardioprotective, gastroprotective, antidiabetic, anti-inflammatory, and antibacterial [10, 11]. *Aronia* fruits are widely utilized to produce natural food pigments and use in nutritional supplements [12, 13].

Anthocyanins are water-soluble pigments and are an important class of flavonoids representing a large group of secondary metabolites found in plant organisms. They are derived from glycosylated polyphenolic compounds and are responsible for the blue, purple, and red color of various plant tissues [14]. Anthocyanins are primarily located in cell vacuoles, and their color is influenced by the intravacuolar environment. There are more than 600 different anthocyanins and 23 anthocyanidins [15, 16]. The six most commonly found anthocyanidins in plants include pelargonidin, delphinidin, peonidin, malvidin, petunidin and cyanidin [17], with the latter found as the most abundant form in *Aronia* [18]. Cyanidin forms found in *Aronia* fruit, from high to low concentration, include cyanidin 3-galactoside (Cy3Gal), cyaniding 3- arbinoside (Cy3A), cyanidin 3-glucoside (Cy3Glu) and cyanidin 3-xyloside (Cy3X), respectively [19].

The flavonoid and anthocyanin biosynthetic pathways have been well characterized in other important fruit crops [20–23]. Flavonoids are synthesized through the phenylpropanoid pathway by converting phenylalanine into 4-coumaroyl-CoA. This reaction is completed by chalcone synthase (*CHS*), from which three different classes of flavonoids are synthesized, including anthocyanins. Leucoanthocyanins are converted by leucoanthocyanidin dioxygenase (*LDOX*/*ANS*) to anthocyanindins and are further glycosylated by uridine diphosphate (UDP)-glucose:flavonoid-O-glycosyl- transferase (*UFGT*) to form cyanidin derivatives [24]. The enzymes involved in flavonoid biosynthesis may be acting as a metabolon that influences the overall efficiency and specificity of the pathway, which also indicates that genes in this pathway may be under concerted transcriptional regulation [25–27]. Transcription factors, including R2R3 *MYB* proteins, basic helix-loop-helix (*bHLH*), and WD40 proteins have essential roles in regulating structural genes in the anthocyanin biosynthetic pathway [24, 28, 29]. The combinations and interactions of these transcription factors regulate the anthocyanin biosynthetic pathway. These structural genes and transcription factors involved in the anthocyanin biosynthetic pathway have been characterized in other important fruit crop species [20, 22, 30, 31].

In the present study, six *A. melanocarpa* accessions were used as the experimental material to establish a database of transcriptome sequences of *Aronia* fruit using Illumina sequencing. The transcriptome and differential expression analysis were used to identify candidate genes involved in anthocyanin biosynthesis between four fruit developmental stages. Transient transformation analyses confirmed the function of AmMYB10 in regulating anthocyanin biosynthesis. The transcriptomic data sets provide a strong foundation for functional studies on *Aronia* and will facilitate breeding improved *Aronia* fruit.

## Results

### Anthocyanin content measurement during fruit development

**Fig. 1:**
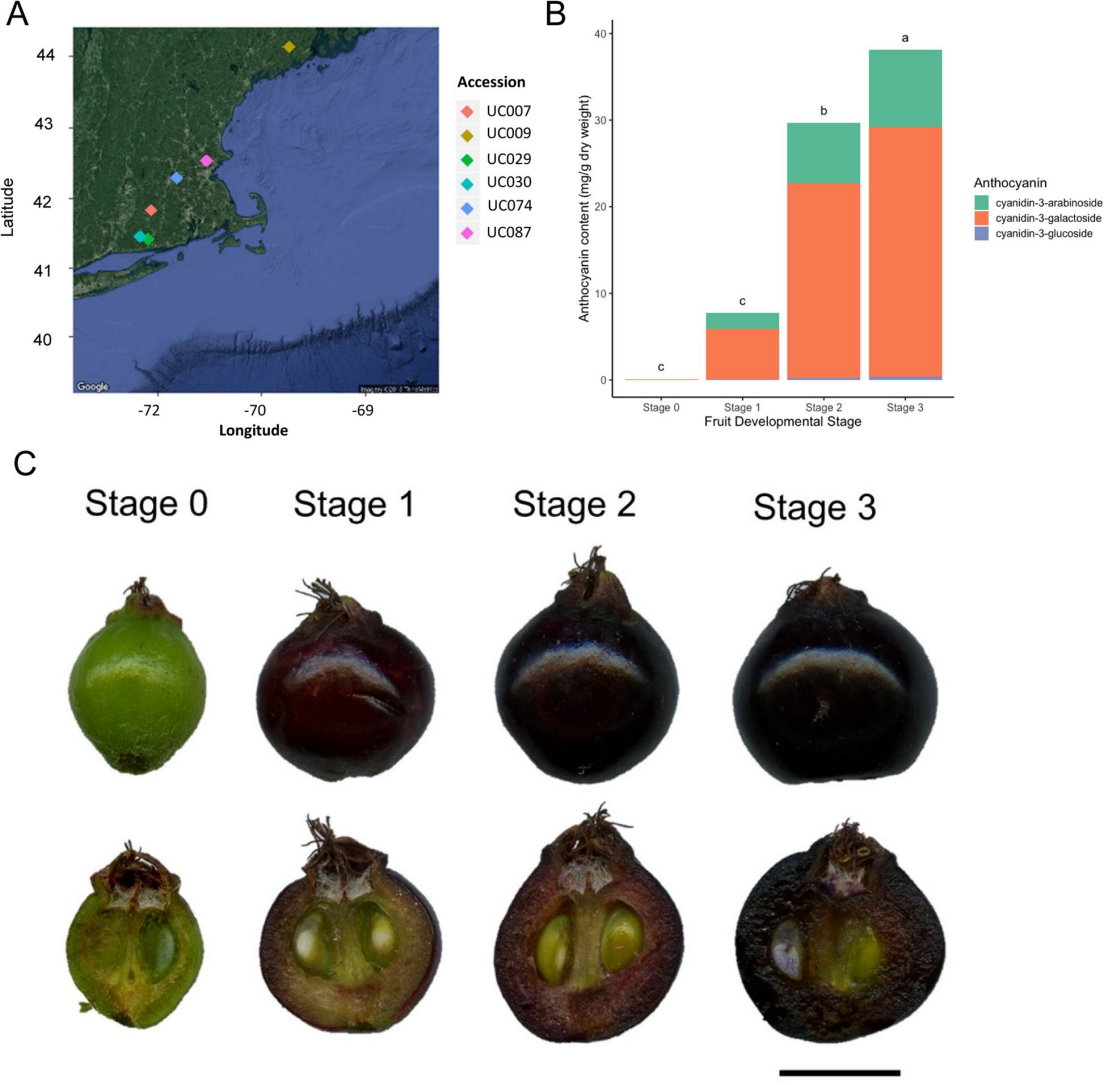
Aronia distribution and anthocyanin accumulation during fruit development. **A** Map of *A. melanocarpa* (2x) accessions. **B** Anthocyanin content and concentration in fruit during four developmental stages. **C** Developmental stages of *A. melanocarpa* fruit. (bar = 5 mm)

Anthocyanin content and concentrations were quantified from the fruit skin of six accessions at four developmental stages. During the progression of fruit development, many fruits will accumulate anthocyanin in their fruits. Consistent with visual appearance, HPLC analysis of fruit skin anthocyanin contents demonstrated significant changes in anthocyanin accumulation profiles between the four developmental stages. The fruits from stage 0 (green fruits) had a total anthocyanin content detected at low levels, only 0.1 mg/g DW. Anthocyanin content increased significantly over the three other developmental stages (Fig. 1A to C). Cy3Gal was the most abundant form of anthocyanin found in *Aronia* fruits. Similar to previous results [8], accession UC009 at stages 2 and 3 had elevated amounts of cyanidin-3-galactoside and reduced cyanidin-3-arabinoside (Supplementary Table S3). This suggests that UC009 has an altered anthocyanin metabolism than other accessions.

### De novo transcriptome assembly and functional annotation

A total number of 341 million trimmed reads were generated from the 24 samples. 7.6, 8.6, 8.7 and 9.1 million trimmed reads were generated from stages 0, 1, 2 and 3, respectively. Individual transcriptome assemblies were generated for each of the 24 samples. The number of transcripts observed in stage 0, 1, 2 and 3 were 538,868 (N50 of 1226), 571,740 (N50 of 1256), 530,617 (N50 of 1291) and 569,236 (N50 of 1220), respectively (Table 1). The de novo assembly produced 90,008 unigenes and an N50 value of 1695 bp (Supplemental Figure S1A). BUSCO analysis determined the de novo transcriptome had 96.1% complete, 2.3% fragmented and 1.6% missing BUSCOs (Supplementary Figure S1B). Overall, we considered the quality of the RNA-seq dataset to be appropriate for further analysis.

**Table 1:** Summary of sequence assembly after sequencing

Sample	Raw seqs	Trimmed seqs	Transcripts	GC %	N50 (bp)	Avg. contig
UC007-0	16,266,511	12,918,578	94,932	43.52	1503	1132.5
UC007-1	20,523,368	16,885,640	107,934	43.30	1715	1251.0
UC007-2	20,677,209	17,257,834	99,253	43.36	1683	1248.0
UC007-3	19,680,123	16,357,270	106,317	43.12	1747	1283.9
UC009-0	17,402,118	14,115,131	98,447	43.20	1734	1284.3
UC009-1	19,044,510	16,612,439	101,483	43.24	1754	1291.3
UC009-2	20,715,687	17,388,235	95,429	43.03	1934	1402.1
UC009-3	20,401,858	17,180,983	97,785	43.28	1761	1047.0
UC029-0	11,552,880	8,497,642	72,564	43.61	1571	1186.4
UC029-1	17,455,593	14,315,974	96,771	43.43	1660	1237.5
UC029-2	17,942,511	14,117,604	81,263	43.80	1630	1226.7
UC029-3	19,572,025	15,929,256	91,572	43.35	1758	1297.1
UC030-0	18,533,052	14,583,810	92,921	43.51	1644	1232.0
UC030-1	15,903,498	11,840,627	86,082	43.57	1633	1221.3
UC030-2	17,798,527	14,172,948	83,011	43.52	1713	1278.1
UC030-3	17,339,014	13,199,343	88,523	43.38	1685	1251.4
UC074-0	14,187,995	11,012,747	85,411	43.68	1624	1206.4
UC074-1	18,222,991	14,154,735	94,404	43.36	1714	1276.3
UC074-2	17,690,591	13,470,011	88,069	43.40	1786	1321.9
UC074-3	20,181,509	16,249,193	103,761	43.33	1676	1242.1
UC087-0	19,237,584	14,871,087	94,593	43.42	1774	1317.1
UC087-1	17,552,575	12,980,271	85,066	43.65	1685	1262.2
UC087-2	15,193,808	10,912,624	83,592	43.57	1705	1271.5
UC087-3	15,923,162	12,724,151	81,278	43.71	1602	1201.4
**Average**	17,874,946	14,239,506	92,103	43.43	1695	1249

In this present study, a total of 36.6% of the 90,008 assembled *Aronia* unigenes were annotated to NCBI Plant Protein database or UniProtKB/Swiss-Prot database and 36% were annotated to the *Arabidopsis* protein database with a gene family assignment and/or similarity search. Based on the NCBI Plant Protein Database and UniProtKB/Swiss-Prot database, of the 19,524 unigenes with a similarity alignment, the *Aronia* unigenes were homologous to sequences in other species. The highest similarity match was *Arabidopsis thaliana* (30%), followed by *Malus* × *domestica* (26.5%), *Pyrus* × *bretschneideri* (22.8%) and *Prunus avium* (1.3%) (Supplementary Figure S2).

The 20,804 unigenes with sequence homology with 12,496 *Arabidopsis* proteins were subjected to GO assignments under biological processes, cellular component and molecular function categories using PlantRegMap. A total of 20,756 unigenes were assigned to at least one GO term (Supplementary Table S4 and S5; Supplementary Figure S3). In the category of biological processes, unigenes related to cellular process, metabolic process, response to stimulus, biological regulation and developmental process were predominant. In molecular functions, genes involved catalytic activity, binding, transporter activity and transcription regulator activity were abundantly expressed (Supplementary Figure S3).

In cellular components, genes related to cell, membrane and protein-containing complex were the most abundant classes. KOG is a database of orthologous eukaryotic genes. A total of 22,328 unigenes were annotated using the KOG database and assigned to 25 classifications (Supplementary Table S6). The most abundant identified classes were signal transduction (14.0%), followed by general function (13.7%), post-translational modification, protein turnover and chaperones (11.4%), translation, ribosomal structure and biogenesis (7.9%), function unknown (6.0%), transcription (7.8%), and intracellular trafficking, secretion, and vesicular transport (6.9%) (Supplementary Table S6).

### Identification of differently expressed genes (DEGs) functional categorization

**Fig. 2:**
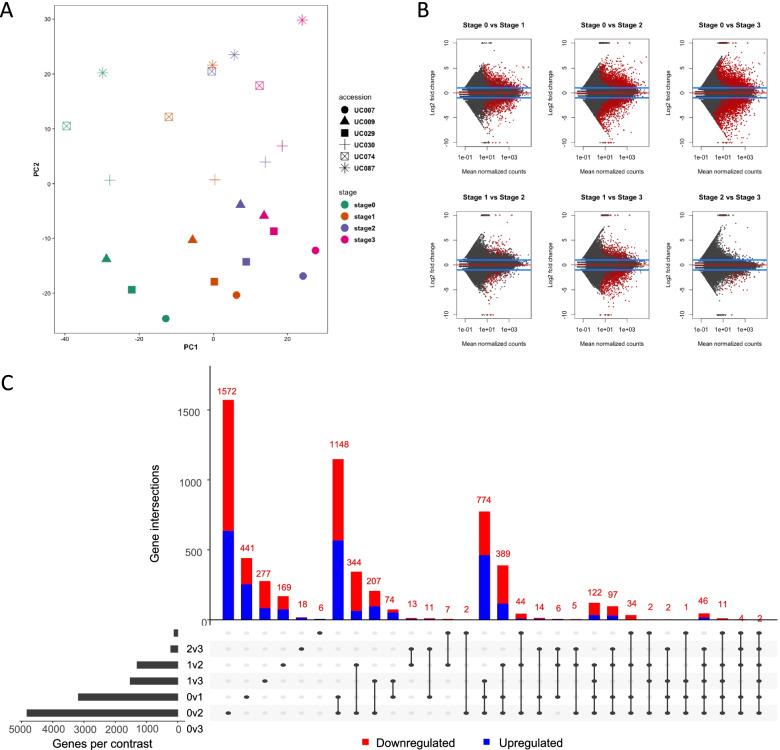
Analysis of transcriptome data from *A. melanocarpa* fruit at four developmental stages. **A** Principal component analysis of the transcriptome data. **B** MA plots for pairwise differential expression analysis contrasts between the four developmental stages. Points in red are significantly differentially expressed (adjusted *P* < 0.05). **C** UpSetR plot of significantly differentially expressed genes (adjusted *P* < 0.05; log2FoldChange > 2)

To identify DEGs during *Aronia* fruit development, we analyzed the transcriptome data at four developmental stages. Principal component analysis (PCA) of the DEGs revealed that the developmental stage was a key factor affecting gene expression in all six accessions, accounting for 20.2% of the observed variance (Fig. 2A). In addition, a genotype effect was also observed, explaining about 15.3% of the variance. Six pairwise contrasts between the four developmental stages were performed with DESeq2 [32] and unigenes were considered differentially expressed using an adjusted *P*-value < 0.05 and absolute log2 fold changes ≥ 1 (Fig. 2B). A total of 5,799 unique unigenes were identified as differentially expressed between the four developmental stages. The DEGs identified in a series of six pairwise comparisons between the four developmental stages 0v1, 0v2, 0v3, 1v2, 1v3 and 2v3 were 277, 441, 1572, 18, 169 and 6, respectively (Fig. 2C).

**Fig. 3:**
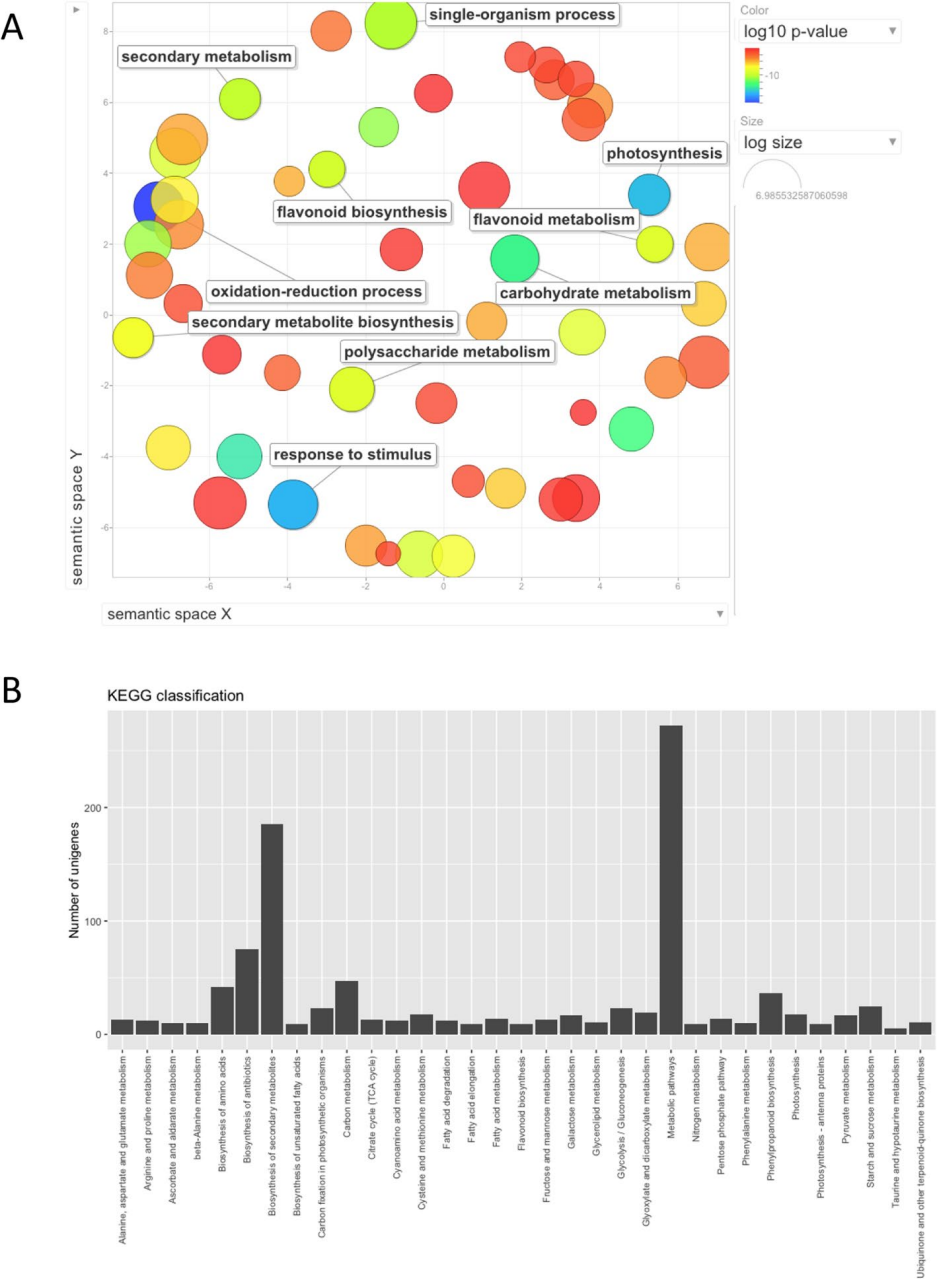
Functional classification of differentially expressed genes. **A** REVIGO Visualization of biological process GO term enrichment analysis of DEGs. GO term enrichment was based on PlantRegMap analysis using Fisher’s exact tests. Each circle represents a term with *P*-value < 0.05. The proximity of terms represents their semantic similarities and the size of the circle represents the size of the term based on Arabidopsis term sizes. The color represents the *P*-value as calculated by PlantRegMap. **B** KOG classification. Bars represent the numbers of unigenes assigned into 26 KOG classes. **C** KEGG classification. Bars represent the numbers of unigenes assigned into 33 KEGG terms

Functional annotation of DEGs was conducted using the *Aronia* differentially expressed protein sequences and blasting them against the *Arabidopsis* TAIR10 protein database (Supplementary Table S6) and Plant Protein database (Supplementary Table S7). Some of the significantly enriched GO terms under biological process included flavonoid biosynthetic process, flavonoid metabolism, secondary metabolism, response to stimulus and photosynthesis (Fig. 3A). REVIGO analysis revealed a large cluster of GO terms associated with flavonoid biosynthesis, suggesting that genes involved with anthocyanin metabolism are significantly enriched. To identify the active biological pathways in *Aronia* fruit, KEGG pathways were used to analyze the pathway annotations of unigene sequences. 1,022 unigenes were assigned to 33 biological pathways in DAVID (Supplementary Table S8). These predicted pathways are responsible for the development of biological compounds. Significant (*P*-value < 0.05) pathways included biosynthesis of secondary metabolites (185 unigenes), phenylpropanoid biosynthesis (36 unigenes), flavonoid biosynthesis (9 unigenes), starch and sucrose metabolism (25 unigenes), and fructose and mannose metabolism (13 unigenes). These results suggest that intensive metabolic activities occur during fruit development and pigmentation in *Aronia* fruit (Fig. 3B). A total of 2,735 unigenes were annotated using the KOG database and assigned to 25 classifications. Frequently identified classes included general function (15.8%), followed by signal transduction mechanisms (14.8%), post-translational modification, protein turnover and chaperones (10.3%), carbohydrate transport and metabolism (9.3%), secondary metabolites biosynthesis, transport and catabolism (8.2%), lipid transport and metabolism (7.0%), and transcription (6.0%) (Supplementary Table S9).

### Clustering of unigenes by expression pattern

**Fig. 4:**
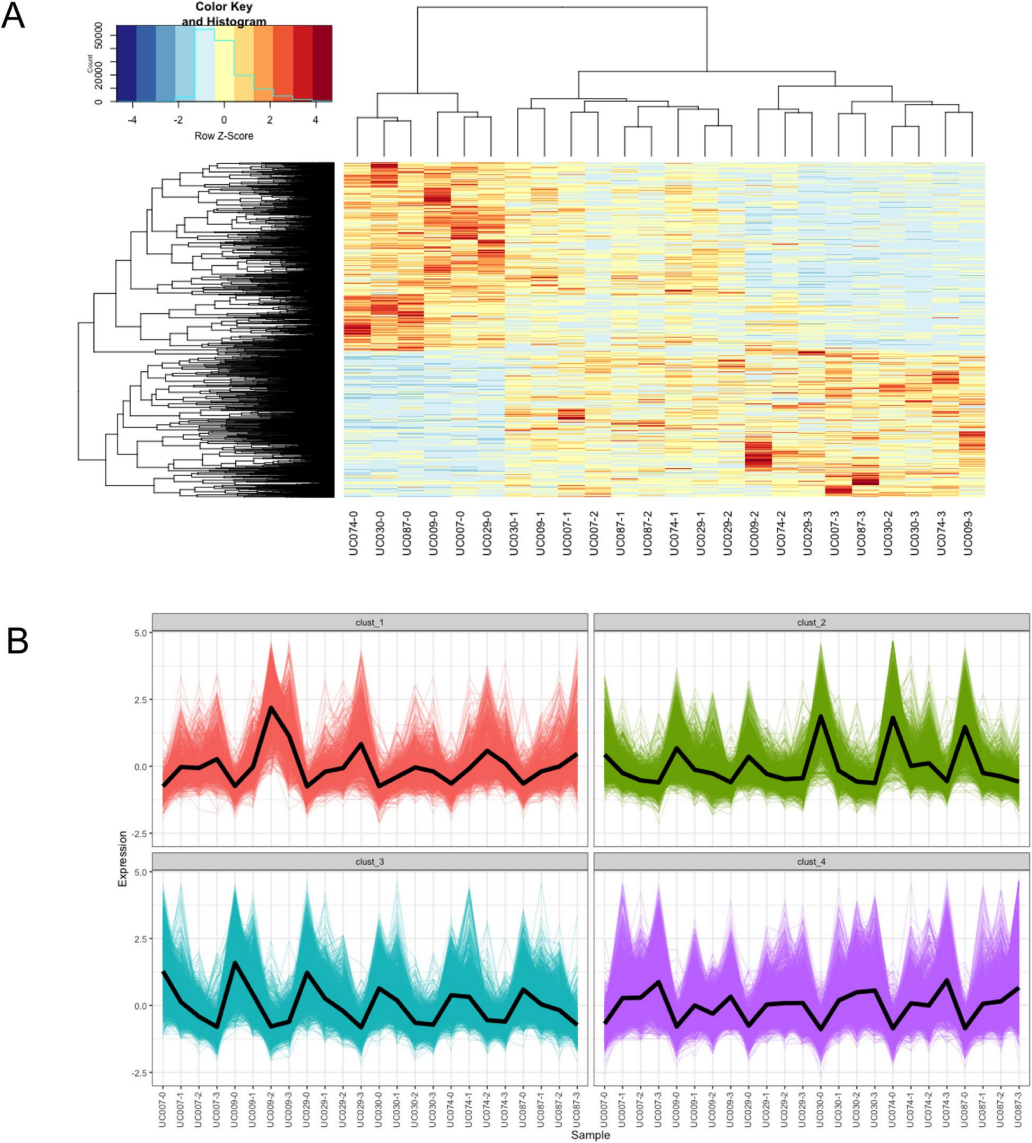
Clustering analysis of gene expression profiles during fruit developmental stages of *A. melanocarpa*. **A** Heat map illustrating the expression profiles of the DEGs. **B** Cluster analysis of DEGs with the K-means method

Fruit development is a complex process that involves synchronized regulation of different metabolic pathways, including hormonal regulation [33], pigmentation [33], sugar metabolism [34] and cell wall metabolism [35]. A set of genes with similar expression patterns tend to be functionally correlated. In this study, novel candidate genes whose functions correlate with the development of different tissues were selected. All 5,799 DEGs were clustered with the hierarchical and K-means method in R software (Supplementary Fig. 4). A heatmap was created to illustrate the variations of gene expression in each tissue (Fig. 4A). As expected, the overall expression of biological replicates was clustered together with similar expression profiles, indicating the reliability of sample collection and analysis.

A total of four clusters was determined as the optimal number of K-means clusters based on the sum of the squared error test (Fig. 4B and Supplementary Figure S4). The unigenes that were located within the same cluster had similar expression patterns during fruit development. Cluster 1 and 4 showed up-regulation between the four developmental stages, whereas clusters 2 and 3 showed down-regulation. These data reveal that major expression profiles are produced by the dynamic and coordinated transitions in mRNA abundance.

### Genes involved in anthocyanin biosynthesis identified from the *Aronia* transcriptome

**Fig. 5:**
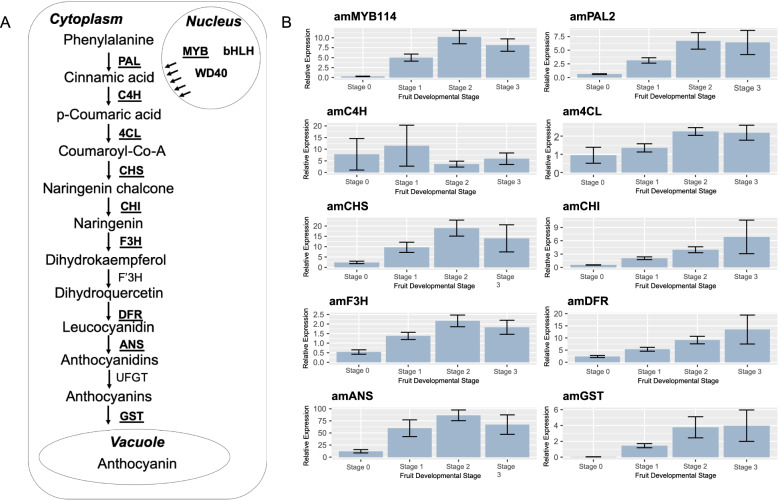
**A** Diagram of the anthocyanin biosynthetic pathway. DEGs are highlighted in bold and validated candidate genes are underlined. **B** Real-time qRT-PCR analysis of expression of structural and regulatory genes involved with anthocyanin biosynthesis at four developmental stages. Bars represent standard error

Anthocyanin biosynthesis is part of the flavonoid biosynthetic pathway in secondary metabolism. Previous studies have shown that fruit anthocyanin content is correlated with the expression of anthocyanin structural and regulatory genes in apple [36, 37], pear [38], and grape [39]. A detailed diagram of the anthocyanin biosynthetic pathway has been illustrated in Fig. 5A. This study identified DEGs involved with the anthocyanin biosynthetic pathway by annotating the assembled unigenes with the *Arabidopsis* protein database and NCBI Plant Protein database against the KEGG database (Supplementary Figure S5). Most genes in the pathway had more than one unique sequence annotated as encoding the same enzyme. Some of the genes in the flavonoid pathway were not found as differentially expressed, possibly due to the genetic diversity between biological replicates. Expression levels of the unigenes showed distinct patterns between the four developmental stages and typically displayed significant upregulation during the ripening process, particularly when the fruit reached stages 2 and 3 (Supplementary Table S10). The expression of these genes might be required for *Aronia* pigmentation because anthocyanin composition is responsible for changes in fruit color.

Phenylalanine ammonia-lyase 2 (*AmPAL2*; TRINITY_DN14080_c0_g1_i5.p1), cinnamate acid 4-hydroxylase (*AmC4H*; TRINITY_DN15961_c0_g1_i3.p1) and 4-coumarate-CoA ligase (*Am4CL*; TRINITY_DN14279_c2_g1_i5.p1) produce the substrate for chalcone synthase (*AmCHS*; TRINITY_DN15104_c0_g1_i3.p2). The initiation of anthocyanin biosynthesis begins with the *CHS* enzymatic catalysis forming naringenin chalcone [40]. Chalcone isomerase (*AmCHI*; TRINITY_DN11846_c0_g1_i1.p3) isomerizes naringenin chalcone to form a flavanone, naringenin [41]. Flavanone-3-hydroxylase (*AmF3H*; TRINITY_DN10705_c0_g1_i1.p2) catalyzes the hydroxylation of flavanones to dihydrokaempferols which are intermediates in the biosynthesis of flavonols, anthocyanidins, catechins and proanthocyanidins. Flavonoid 3’-hydroxylase (*F3’H*) further hydroxylates dihydrokaempferols at the 3’ and 5’ ends of the B-ring to produce dihyroquercetin [42]. Dihydroflavonol 4-reductase (*AmDFR*; TRINITY_DN10073_c0_g1_i3.p1) uses nicotinamide adenine dinucleotide phosphate (NADPH) as a cofactor to catalyze the stereo specific reduction of dihydroflavonols to leucoanthocyanidins [43]. Subsequently, anthocyanidin synthase (*AmANS*; TRINITY_DN17487_c0_g2_i1.p1) converts leucoanthocyanidins into anthocyanidins [44]. The unstable free hydroxyl group at the 3 position of the heterocyclic ring is stabilized by UDP-glucose: flavonoid-3-O-glycosyltransferase (*UFGT*) forming anthocyanins and allowing them to accumulate in the vacuoles as water-soluble pigments. Previous studies have shown that the *UFGT* enzyme has been the key regulatory step in anthocyanin biosynthesis in pear [45] and is critical for fruit pigmentation [31, 46]. However, there appears to be a pattern of most genes in the anthocyanin pathway functioning together to control anthocyanin biosynthesis. In the present study, we did not find the *UFGT* gene involved with anthocyanin biosynthesis to be differentially expressed, indicating that *UFGT* transcript level is not a limiting factor in Aronia. After synthesis of the anthocyanins, glutathione S-transferase (GST) proteins physically bind to anthocyanins and act as anthocyanin carrier proteins that facilitate transport from the cytoplasm to the vacuole [47]. *AmGST* (TRINITY_DN13448_c1_g3_i5.p1) had suppressed expression during stage 0 (green fruit) but significantly increased over the three other developmental stages (Supplementary Table S10).

### Realtime RT-PCR and correlation analysis of genes involved in anthocyanin biosynthesis

Among all the identified DEGs, those unigenes involved in anthocyanin biosynthesis were closely related to the observed changes in fruit pigmentation during developmental stages. Twelve of these genes were selected for qRT-PCR analysis (Fig. 5B and Supplementary Fig. 5). The real-time qRT-PCR results were, for the most part, consistent with those obtained from DEG analysis (Fig. 5B). However, as shown from the results of differential expression analysis, the real-time qRT-PCR assay shows a significant amount of variation between different accessions, which may be resulted from genetic variation among the accessions collected from the wild.

**Fig. 6:**
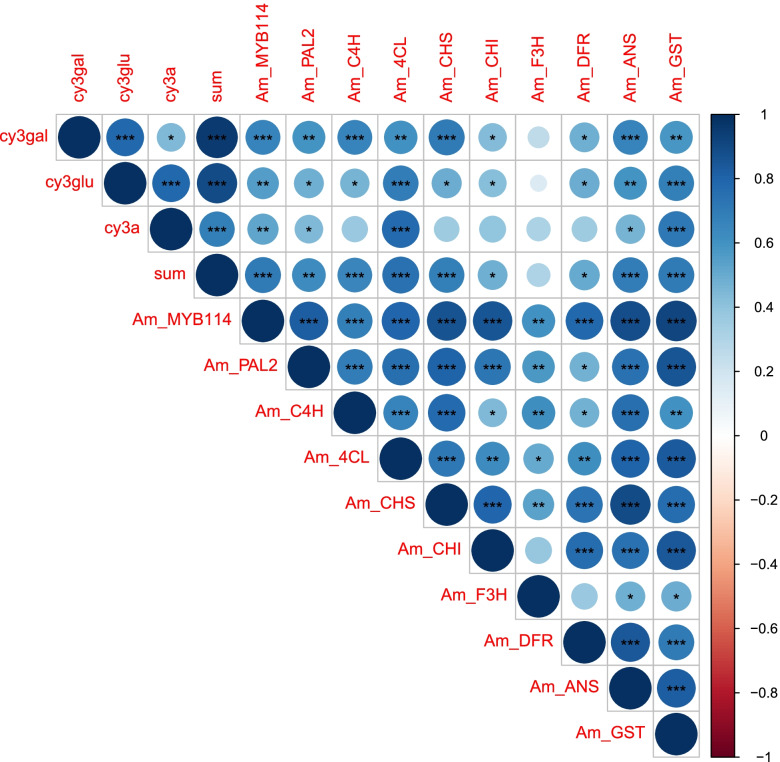
Correlation matrix of cyanidin derivatives and gene expression of candidate genes involved with anthocyanin biosynthesis

For the anthocyanin biosynthesis genes, we performed a correlation analysis and found a significant correlation between most candidate genes related to cy3gal, cy3glu, cy3a and total anthocyanin (Fig. 6). *AmF3H* did not have a significant correlation with any of the cyanidin derivatives. The lack of correlation could be cause by the expression pattern of *AmF3H*, which exhibited an increase in expression from stage 0 to stage 1, but then plateaued (Supplementary Figure S3). Several genes, including *AmPAL2*, *Am4CL*, *AmANS* and *AmGST*, showed a significant positive correlation with all cyanidin derivatives. The strong positive correlation provides further evidence that the flavonoid pathway is functioning together to control anthocyanin biosynthesis.

### Identification of MYB10 as a regulator of anthocyanin biosynthesis

Transcription factors (TFs) have essential roles in regulating secondary metabolism by orchestrating the expression of genes in biosynthetic pathways [48]. There were 272 TF encoding unigenes among the DEGs identified through Aronia fruit development. Several of these TFs were classified into the bHLH (28), ERF (26), MYB (25) and WRKY (15) families (Supplementary figure S6). These groups of TFs may regulate different metabolic pathways, such as in phenylpropanoid and flavonoid biosynthesis.

**Fig. 7:**
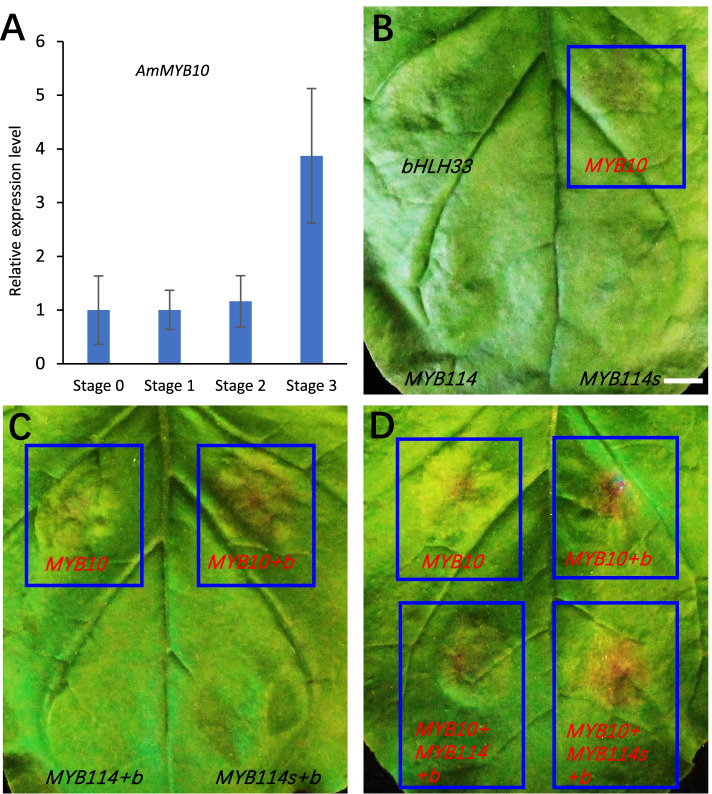
Expression and transient transformation assays identify AmMYB10 as an activator of anthocyanin biosynthesis leaves. **A** Real-time RT-PCR analysis showing increased expression of AmMYB10 during fruit development. Expression of *AmACTIN* was used as a reference gene. Error bars are standard error of three independent biological replicates. **B** A representative *Nicotiana benthamiana* leaf image infiltrated with AmbHLH33, AmMYB10, AmMYB114 and AmMYB114s agrobacterium culture. Noting weak anthocyanin accumulation after AmMYB10 infiltration. **C** Combination of AmMYB10 with AmbHLH33 (MYB10 + b) enhances anthocyanin accumulation, while the combination of AmbHLH33 with either AmMYB114 or AmMYB114s does not activate anthocyanin biosynthesis. **D** The combination of AmMYB10 with AmMYB114 or AmMYB114s does not affect anthocyanin accumulation. Digital photographs were taken 6 days after infiltration. Bar = 2 cm

Previous studies indicated that TFs in the MYB, bHLH and WD40 proteins can form MBW protein complexes in regulating structural genes in the anthocyanin biosynthetic pathway [49]. In this study, we have identified DEGs annotated as R2R3 type MYB TFs involved with anthocyanin biosynthesis. Among these DEGs, TRINITY_DN12211_c0_g1_i1.p1 has close homology to *AtMYB75* and *PyMYB114* (*Pyrus* x *bretschneideri*), which has been shown to regulate fruit anthocyanin biosynthesis [50]. The expression of the *AmMYB114* transcription factor significantly increased during the fruit development process (Fig. 5B and Supplementary Table S10). Another MYB gene, TRINITY_DN11093_c1_g2_i1.p1, had close homology to a putative *P.* x *bretschneideri* MYB transcription factor, although the function of this gene has not been reported. The *AmMYB10* (TRINITY_DN10934_c2_g5_i4.p1), a homolog of the *pyMYB10*, also showed a slightly increased expression level, which was also confirmed with real-time qRT-PCR using samples from different fruit developmental stages (Fig. 7A).

To identify and characterize the biological function of the MYBs in regulating anthocyanin biosynthesis in Aronia, we selected the AmMYB114 and AmMYB10 as candidates according to the transcriptome data. Further, paralogs of these two MYBs regulate flavonoid biosynthesis in *Rosaceae* family species [38, 45, 50, 51]. To study the biological function of these two *AmMYB*s, we employed transient expression assays using leave infiltration in *Nicotiana benthamiana*. The coding sequences of *AmMYB114* and *AmMYB10* were cloned using PCR based approach with gene-specific primers (Supplementary Table S2). Interestingly, the *AmMYB114* gene has two alternative splicing forms, as confirmed by sanger sequencing. The shorter splicing form of the *AmMYB114* gene (*AmMYB114s*) is 104 bp shorter due to an additional intron removed from the middle of the *AmMYB114* coding sequence, which also causes a switch in the reading frame. The AmMYB114s protein shares 151 amino acids identical to the N-terminal region of the AmMYB114 protein. The AmMYB114s would have an intact MYB domain responsible for DNA binding but without a transcriptional activation domain, which raises the possibility of competing for DNA binding with the AmMYB114.

These three MYB genes, e.g., *AmMYB10*, *AmMYB114* and *AmMYB114s*, were cloned into the destination vector and transformed into agrobacterium. We cloned *AmbHLH33* from *Aronia* based on sequence homology with MdbHLH33, which interacts with MdMYB10 in *Malus domestica* [52]. Infiltration experiments using *N. bethaminana* leaves indicated that AmMYB10 resulted in weak anthocyanin accumulation, indicating activation of anthocyanin biosynthesis (Fig. 7B). We did not detect anthocyanin accumulation in leaves transformed with AmbHLH33, AmMYB114, or AmMYB114s in the transient infiltration assays (Fig. 7B). Combining AmbHLH33 with AmMYB10 enhanced anthocyanin accumulation, indicating that these two proteins may function together in regulating anthocyanin biosynthesis (Fig. 7C). Previous studies reported that PyMYB114 enhance the function of PyMYB10 in Pyrus [50]. In this study, co-transformation of AmMYB114 together with AmMYB10 did not enhance the function of AmMYB10 (Fig. 7D). The AmMYB114s did not show activation function in the transient transformation assay, and it didn’t interfere with the activation function of AmMYB10 neither (Fig. 7D). These experiments indicate that AmMYB10 functions as a bona fide activator of anthocyanin biosynthesis in *Aronia*.

## Discussion

This study reports the transcriptome analysis of Aronia fruit development using Illumina RNA-sequencing. The *Aronia* de novo transcriptome with annotation provides a new valuable resource for studying fruit development in *Aronia*. In this research, a total number of 341 million trimmed reads were generated from the 24 samples. These data especially facilitate the discovery of novel genes in the metabolism and ripening process of *Aronia* fruits and might also be helpful for studies in closely related species. About 36% of the Aronia unigenes were annotated to either NCBI Plant Protein database or the Arabidopsis protein database, but approximately 63% of unigenes were not annotated. We reasoned this might be either due to non-coding regions or caused by the inadequate length of sequences or the lack of information in databases. The Go analysis indicated that the 20,804 unigenes with sequence homology with 12,496 Arabidopsis proteins were in biological processes, cellular component and molecular function categories.

In the pairwise comparison between the four developmental stages using DESeq2 (Love, Huber, and Anders 2014), we identified a total of 5,799 unique unigenes as differentially expressed genes. A large number of DEGs are involved in fruit development and a group of key genes involved with anthocyanin biosynthesis. Unigenes that code for enzymes from pathways, such as flavonoid biosynthesis, phenylpropanoid biosynthesis that control pigmentation changes during fruit development were identified in this research. None of these candidate genes have been studied or reported to be involved in anthocyanin biosynthesis in *Aronia*. Future studies will be needed to determine the biological function of these candidate genes.

Additionally, the interaction and relationships between candidate structural genes and transcription factors will need to be investigated. Nevertheless, this research has provided a valuable resource for investigating particular biological processes in fruit development. We do observe a significant amount of expression variation between biological replicates. This could contribute to the biological variation between the replicates. The germplasms were collected from the wild and was grown out in a controlled field setting. Therefore, the genetic variability may be contributing to the variability observed in the expression data.

Anthocyanin biosynthetic pathways are regulated by MYB-bHLH-WD repeat (MBW) complexes [28, 49]. In our transcriptome analysis, AmMYB114 is the one of the most significantly upregulated MYB genes. The paralogs of AmMYB114, such as AtMYB75 in Arabidopsis and PyMYB114 in Pyrus [50], are regulators of anthocyanin biosynthesis in fruit. In this research, we selected AmMYB114 as our major candidate at the beginning. However, the AmMYB114 did not activate anthocyanin biosynthesis in our transient transformation assays, which were performed meticulously and repeated many times. Surprisingly, the AmMYB10, a mildly upregulated MYB gene during the fruit ripening process (Fig. 7A), is necessary and sufficient to activate anthocyanin biosynthesis (Fig. 7B-D). The function of AmMYB10 is enhanced when co-expressed with AmbHLH33 (Fig. 7C), which is consistent with previous reports in apple and pear [52]. In contrast, AmMYB10 did not show a synergistic effect with AmMYB114 in the transient transformation assays, which is different from its paralogs [28, 50]. These experiments indicated that the regulation of Aronia anthocyanin accumulation may have differentiated from other plants in the Rosaceae family.

The AmMYB114s is an alternative splicing form and truncated version of the AmMYB114, with an intact MYB domain but no activation domain. In theory, the AmMYB114s may compete with AmMYB114 protein for DNA binding, therefore affecting the normal function of AmMYB114. In this study, AmMYB114 does not activate anthocyanin biosynthesis and its function in fruit development remains to be discovered. It would be interesting to test if AmMYB114s would interfere with the function of AmMYB114 once the biological function of AmMYB114 has been determined. In this study, the function of AmMYB10 was not affected by co-expression with AmMYB114 or AmMYB114s, further confirming that AmMYB114 may be involved in regulating a different biosynthetic pathway during fruit development. In summary, we have identified AmMYB10 as a functional positive regulator in anthocyanin biosynthesis in *Aronia* fruit.

## Methods

### Plant material and RNA extraction

*Aronia melanocarpa* fruits from six accessions (Fig. 1A; Supplementary Table S1) at four different stages of development (Fig. 1B) were harvested from the *Aronia* germplasm collection in Storrs, CT. The Aronia accessions were identified and collected by Dr. Mark Brand and his group. All plant materials are maintained at the UConn Research Farm and are publicly available for request through the USDA-ARS-NCRPIS using the accession numbers in Supplementary Table S1. All methods were performed in accordance with the relevant guidelines and regulations.

Fruits were collected in the field and immediately transferred to the lab for further processing. Fruit skins were removed, frozen in liquid nitrogen and stored at -80 °C until further use. Total RNA from fruit samples were extracted using a modified CTAB method as described [53]. The concentration of extracted RNA was determined using a NanoDrop-1000 spectrophotometer (Thermo Scientific, Willington, DE) and RNA integrity was evaluated using an Aglient 2100 Bioanalyzer (Agilent Technologies, Santa Clara, CA, USA). Only samples with RIN values above 7.0 were used for library preparation. Libraries were constructed at the University of Connecticut Center for Genome Innovation following the TruSeq Stranded Total Library Prep Kit protocol (Illumina, San Diego, CA). Libraries were prepared for the Illumina HiSeq 2500 in High Output mode (2 × 100 bp). A total of 24 libraries were sequenced across three lanes.

### Determination of anthocyanin content

Fruits skin tissue from each of the 24 samples were lyophilized and ground into a powder to measure total anthocyanin content. For each extraction, approximately 30 mg of berry tissue powder was placed into a microcentrifuge tube. Equivolume amounts of 12 mM HCl in ultrapure water and 12 mM HCl in methanol were added to the tubes at a ratio of 20 mg per mL solvent. The sample was mixed on a digital vortex mixer at 3000 rpm for 30 s and sonicated in a 30 ^0^C water bath for 10 min. This mixing and sonicating were repeated twice more to extract anthocyanins. The extract was isolated by centrifuging the sample at 5000 × g for 5 min and decanting the liquid layer into an HPLC vial. Extractions were performed in triplicate. Extracts were analyzed by reverse phase HPLC using a Dionex UltiMate 3000 HPLC equipped with an LPG-3400 quaternary pump, a WPS-3000 analytical autosampler, a DAD- 3000 diode array detector, and an FLD-3100 fluorescence detector, based on a prior method (Dorris et al., 2018). Injections of 10 µL were loaded onto a Kinetex 5 µm EVO C18, 100 Å, 250 X 4.6 mm column (Phenomenex, Torrance, CA, USA) at 15 ^0^C. Anthocyanins were resolved using a binary gradient of formic acid: water (5:95, v:v) (A) and methanol (B). The flow rate was constant at 1 mL/min. The gradient began at 5% B for 1 min, increased to 35% B over 39 min, then to 95% B over 10 min, and remained constant at 95% B for 5 min, then decreased to 5% B over 2 min, and re-equilibrated at 5% B for the remaining 8 min. Absorbance data was collected from 190 to 700 nm, and chromatograms were analyzed at 520 nm to detect anthocyanins. The elution order and spectral characteristics were used to assign the most prominent anthocyanin peaks [19]. Anthocyanin concentration was determined using external calibration over the linear range 1–750 µg cyanidin-3-galactoside equivalents/ mL solution.

### De novo transcriptome assembly and differential expression analysis

Paired-end raw reads were filtered to exclude low-complexity reads and reads containing adaptor sequences. Raw reads were quality controlled and trimmed prior to assembly using Sickle (https://github.com/najoshi/sickle) with a minimum read length of 45 bp and minimum Phred-scaled quality value of 30. The 24 trimmed reads were independently de novo assembled using Trinity [54] with a minimum contig length of 300. TransDecoder [55] was used to determine the optimal open reading frames with homology to known proteins using the PFAM Protein Database [56]. The open reading frames of all transcripts were clustered using USEARCH-UCLUST [57] to find redundancy between the assembled transcripts with a minimum threshold of 80% identity. Trimmed reads from the 24 cDNA libraries were mapped to the assembled unigenes using Bowtie2.

Differential expression analysis of samples was performed using the DESeq2 [32] R package. Unigene expression levels were quantified using the normalized count values from DESeq2. Unigenes differentially expressed between samples were screened using *P*-value < 0.05 and absolute log2 fold changes ≥ 1 as the threshold.

Hierarchical clustering of DEGs was performed in R using Z-scaled FPKM data and clustering based on Pearson’s correlation and complete linkage clustering. Z-scaled FPKM values were grouped by k-means clustering, four clusters were chosen based on the least within-group sum of squares method.

### Validation of de novo assembly by BUSCO

We quantified the completeness of the de novo assembly by comparing the assembled transcript set against a set of highly conserved single-copy orthologs using BUSCO [58]. The number of complete, duplicated and missing fragments were calculated.

### Transcriptome annotation

The assembled de novo transcriptome was annotated with the Eukaryote Non-model Transcriptome Annotation Pipeline (EnTAP) [59]. To investigate the potential functions of *A. melanocarpa* unigenes, we annotated all unigenes using EnTAP with an E-value threshold 10^–5^ using the NCBI Plant Protein database (release 87), UniProtKB/Swiss-Prot database, EggNOG database [60], PFAM Protein database, Gene Ontology (GO) and Kyoto encyclopedia of genes and genomes (KEGG) [61, 62]. EuKaryotic Orthologous Groups (KOG) were determined by searching proteins against the NCBI KOG database using the WebMGA server [63].

In addition, EnTAP was used with an E-value threshold of 10^–3^ and query coverage at least 50% against the *Arabidopsis thaliana* TAIR10 Protein database for functional categorization of unigenes. GO analysis was performed for functional annotation of the entire transcriptome and significantly differentially expressed genes using PlantRegMap software [64]. Our targets are compared with these databases to infer GO annotation at PlantRegMap. KEGG pathway analysis was completed by using TAIR codes in DAVID [65]. Transcription factors (TFs) from the DESeq2 DEGs were identified by matching TAIR codes to the *A. thaliana* transcription factor database (Plant TFDB v5.0) [64].

Pearson correlation coefficients were calculated between FPKM values and anthocyanin concentrations in R for correlation analysis of candidate genes involved with anthocyanin biosynthesis and expression levels.

### qRT-PCR analysis

Twelve unigenes were selected for validation using qRT-PCR. Specific primer pairs for selected genes used in qRT-PCR were designed as shown in Supplementary Table S2. Three micrograms of total RNA were reverse‐transcribed using a Superscript III RT kit (Invitrogen) in a 20 μl reaction system. The cDNA was diluted 50 times and used as the template for qRT–PCR. PCR amplification was performed on an ABI 7900HT real‐time PCR machine using SYBR Green qPCR master mix (Life Technologies). For each reaction, 2 μl diluted cDNA sample was used in a 10 μl reaction system. The PCR reaction program was set according to the manufacturer's instructions (Life Technologies). The *Aronia* reference gene (β-ACTIN) was used for normalization. The comparative CT method (2-ΔΔCT method) was used to analyze the expression levels of the different genes [66].

### Transient transformation of *Nicotiana benthamiana* leaves

*Nicotiana benthamiana* plants were grown to six leaves in a growth chamber with a temperature setting of 22 ℃ and a light cycle of 16 h light/8 h dark. Agrobacterium cultures were incubated in LB medium at 28 ℃ overnight. Cells were collected by centrifugation at 5000 rpm for 10 min and resuspended in infiltration buffer (half-strength MS medium supplemented with 2% sucrose and 200 μM acetosyringone and pH was adjusted to 5.6) with a 0.2 of OD600 value. Agrobacterium with constructs of *AmMYB10*, *AmMYB114*, and *AmMYB114sI*, or combined with the *AmbHLH33*, were infiltrated into *N. benthamiana* leaves and observed for pigmentation after 4 days. Combinations of *AmMYB10* with *AmMYB114* or *AmMYB114s* were also performed to investigate possible synergistic effects between these MYB genes. Digital photographs were taken 6 days after infiltration. To control leaf-to-leaf variabilities, each treatment was repeated at least on three leaves with positive and negative controls.

## Supplementary Information

**Additional file 1:** **Supplementary Table S2.** Primer sequences used for qRT-PCR validation of RNA-seq data and cloning.

**Additional file 2:** **Supplementary Figure S1.** Transcriptome assembly length and quality. **Supplementary Figure S2.** Species distribution of BLAST top hits against the NCBI NR protein database with an E value cut-off of 1e-5. **Supplementary Figure S3.** Assignment of unigenes into different categories based on GO terms. **Supplementary Figure S4.** Clustering of candidate genes based on expression pattern correlated with fruit development. **Supplementary Figure S5.** Expression levels of structural and regulator genes involved with anthocyanin biosynthesis from the fruits of A. melanocarpa (n=6) at four developmental stages. **Supplementary Figure S6.** Transcription factors distribution in different gene families. Counts of transcription factors within the 5,799 differentially expressed genes were identified by searching the top A. thaliana BLASTx hits for TAIR codes within the transcription factor database (Plant TFDB v3.0).

## Data Availability

The datasets generated and analysed during the current study are available in the NCBI BioProject and Sequence Read Archive under the accession number PRJNA603127. Germplasm can be requested from the USDA-ARS-NCRPIS using the accession numbers in Supplementary Table S1.

## References

[1] Hummer KE, Janick J. Rosaceae. Taxonomy, economic importance, genomics. Genet Genomics Rosaceae. 2009;1–17. 10.1007/978-0-387-77491-6_1.

[2] Campbell CS, Evans RC, Morgan DR, Dickinson TA, Arsenault MP (2007). Phylogeny of subtribe Pyrinae (formerly the Maloideae, Rosaceae): Limited resolution of a complex evolutionary history. Plant Syst Evol..

[3] Leonard PJ, Brand MH, Connolly BA, Obae SG (2013). Investigation of the origin of aronia mitschurinii using amplified fragment length polymorphism analysis. HortScience.

[4] Mahoney JD, Hau TM, Connolly BA, Brand MH (2019). Sexual and apomictic seed reproduction in aronia species with different ploidy levels. HortScience.

[5] Brand MH. Aronia: Native shrubs with untapped potential. Arnoldia. 2010;67:14–25. https://www.google.com/search?q=Aronia%3A+Native+Shrubs+With+Untapped+Potential.&sxsrf=ALeKk03qPIokYMlmOoFiWLdzLEp6AlXQbg%3A1628259155006&source=hp&ei=UkMNYdTmOseFwbkPzqSfsAw&iflsig=AINFCbYAAAAAYQ1RY6bnt5oqXNm1Qnu4JsIyiNKVGmxu&oq=Aronia%3A+Native+Shrubs+W. Accessed 6 Aug 2021.

[6] Zheng W, Wang SY (2003). Oxygen radical absorbing capacity of phenolics in blueberries, cranberries, chokeberries, and lingonberries. J Agric Food Chem.

[7] Xianli Wu †, Liwei Gu †, Ronald L. Prior *,†, McKay§ S. Characterization of anthocyanins and proanthocyanidins in some cultivars of ribes, aronia, and sambucus and their antioxidant capacity. J Agric Food Chem. 2004;52:7846–56. 10.1021/JF0486850. 10.1021/jf0486850 15612766

[8] Brand MH, Connolly BA, Levine LH, Richards JT, Shine SM, Spencer LE (2017). Anthocyanins, total phenolics, ORAC and moisture content of wild and cultivated dark-fruited Aronia species. Sci Hortic (Amsterdam).

[9] Babu PVA, Liu D, Gilbert ER (2013). Recent advances in understanding the anti-diabetic actions of dietary flavonoids. J Nutr Biochem.

[10] Kokotkiewicz A, Jaremicz Z, Luczkiewicz M (2010). Aronia plants: a review of traditional use, biological activities, and perspectives for modern medicine. J Med Food.

[11] Jurikova T, Mlcek J, Skrovankova S, Sumczynski D, Sochor J, Hlavacova I, et al. Fruits of black chokeberry aronia melanocarpa in the prevention of chronic diseases. Molecules. 2017;22. 10.3390/molecules22060944. 10.3390/molecules22060944 PMC6152740 28590446

[12] Howard LR, Brownmiller C, Prior RL, Mauromoustakos A (2013). Improved stability of chokeberry juice anthocyanins by β-cyclodextrin addition and refrigeration. J Agric Food Chem.

[13] Sikora J, Broncel M, Markowicz M, Chałubiński M, Wojdan K, Mikiciuk-Olasik E (2012). Short-term supplementation with Aronia melanocarpa extract improves platelet aggregation, clotting, and fibrinolysis in patients with metabolic syndrome. Eur J Nutr.

[14] Tanaka Y, Sasaki N, Ohmiya A (2008). Biosynthesis of plant pigments: anthocyanins, betalains and carotenoids. Plant J.

[15] Castañeda-Ovando A, de Pacheco-Hernández M L, Páez-Hernández ME, Rodríguez JA, Galán-Vidal CA (2009). Chemical studies of anthocyanins: A review. Food Chem.

[16] Wallace TC, Giusti MM (2015). Anthocyanins. Adv Nutr.

[17] Khoo HE, Azlan A, Tang ST, Lim SM. Anthocyanidins and anthocyanins: colored pigments as food, pharmaceutical ingredients, and the potential health benefits. Food Nutr Res. 2017;61. 10.1080/16546628.2017.1361779. 10.1080/16546628.2017.1361779 PMC5613902 28970777

[18] Slimestad R, Torskangerpoll K, Nateland HS, Johannessen T, Giske NH (2005). Flavonoids from black chokeberries Aronia melanocarpa. J Food Compos Anal.

[19] Taheri R, Connolly BA, Brand MH, Bolling BW (2013). Underutilized chokeberry (Aronia melanocarpa, Aronia arbutifolia, Aronia prunifolia) accessions are rich sources of anthocyanins, flavonoids, hydroxycinnamic acids, and proanthocyanidins. J Agric Food Chem.

[20] Feng C, Chen M, Xu C, Bai L, Yin X, Li X (2012). Transcriptomic analysis of Chinese bayberry (Myrica rubra) fruit development and ripening using RNA-Seq. BMC Genomics.

[21] Crifò T, Puglisi I, Petrone G, Recupero GR, Lo Piero AR (2011). Expression analysis in response to low temperature stress in blood oranges: Implication of the flavonoid biosynthetic pathway. Gene.

[22] El-Sharkawy I, Liang D, Xu K (2015). Transcriptome analysis of an apple (Malus × domestica) yellow fruit somatic mutation identifies a gene network module highly associated with anthocyanin and epigenetic regulation. J Exp Bot.

[23] Hyun TK, Lee S, Rim Y, Kumar R, Han X, Lee SY (2014). De-novo RNA Sequencing and Metabolite Profiling to Identify Genes Involved in Anthocyanin Biosynthesis in Korean Black Raspberry (Rubus coreanus Miquel). PLoS One.

[24] Jaakola L (2013). New insights into the regulation of anthocyanin biosynthesis in fruits. Trends Plant Sci.

[25] Yuan Y, Chiu LW, Li L (2009). Transcriptional regulation of anthocyanin biosynthesis in red cabbage. Planta.

[26] Qi X, Shuai Q, Chen H, Fan L, Zeng Q, He N (2014). Cloning and expression analyses of the anthocyanin biosynthetic genes in mulberry plants. Mol Genet Genomics.

[27] Jørgensen K, Rasmussen AV, Morant M, Nielsen AH, Bjarnholt N, Zagrobelny M, et al. Metabolon formation and metabolic channeling in the biosynthesis of plant natural products. Curr Opin Plant Biol. 2005;8(3) SPEC. ISS.:280–91. 10.1016/j.pbi.2005.03.014. 10.1016/j.pbi.2005.03.014 15860425

[28] Petroni K, Tonelli C (2011). Recent advances on the regulation of anthocyanin synthesis in reproductive organs. Plant Sci.

[29] Grotewold E (2005). Plant metabolic diversity: a regulatory perspective. Trends Plant Sci.

[30] Carbone F, Preuss A, De Vos RCH, D’Amico E, Perrotta G, Bovy AG (2009). Developmental, genetic and environmental factors affect the expression of flavonoid genes, enzymes and metabolites in strawberry fruits. Plant, Cell Environ.

[31] Liu H, Su J, Zhu Y, Yao G, Allan AC, Ampomah-Dwamena C, et al. The involvement of PybZIPa in light-induced anthocyanin accumulation via the activation of PyUFGT through binding to tandem G-boxes in its promoter. Hortic Res. 2019;6:1–13. 10.1038/s41438-019-0217-4. 10.1038/s41438-019-0217-4 PMC6885052 31814987

[32] Love MI, Huber W, Anders S. Moderated estimation of fold change and dispersion for RNA-seq data with DESeq2. Genome Biol. 2014;15:1–21. 10.1186/S13059-014-0550-8. 10.1186/s13059-014-0550-8 PMC4302049 25516281

[33] Barry CS, Giovannoni JJ (2006). Ripening in the tomato Green-ripe mutant is inhibited by ectopic expression of a protein that disrupts ethylene signaling. Proc Natl Acad Sci.

[34] Oms-Oliu G, Hertog MLATM, Van de Poel B, Ampofo-Asiama J, Geeraerd AH, Nicolai BM (2011). Metabolic characterization of tomato fruit during preharvest development, ripening, and postharvest shelf-life. Postharvest Biol Technol.

[35] Carrari F, Baxter C, Usadel B, Urbanczyk-Wochniak E, Zanor M-I, Nunes-Nesi A (2006). Integrated analysis of metabolite and transcript levels reveals the metabolic shifts that underlie tomato fruit development and highlight regulatory aspects of metabolic network behavior. Plant Physiol.

[36] Feng F, Li M, Ma F, Cheng L (2013). Phenylpropanoid metabolites and expression of key genes involved inanthocyanin biosynthesis in the shaded peel of apple fruit in response to sun exposure. Plant Physiol Biochem.

[37] Liu Y, Che F, Wang L, Meng R, Zhang X, Zhao Z (2013). Fruit coloration and anthocyanin biosynthesis after bag removal in non-red and red apples (Malus × domestica Borkh.). Molecules.

[38] Feng S, Wang Y, Yang S, Xu Y, Chen X (2010). Anthocyanin biosynthesis in pears is regulated by a R2R3-MYB transcription factor PyMYB10. Planta.

[39] Soubeyrand E, Basteau C, Hilbert G, Van Leeuwen C, Delrot S, Gomès E (2014). Nitrogen supply affects anthocyanin biosynthetic and regulatory genes in grapevine cv. Cabernet-Sauvignon berries Phytochemistry.

[40] Sun W, Meng X, Liang L, Jiang W, Huang Y, He J (2015). Molecular and biochemical analysis of chalcone synthase from freesia hybrid in flavonoid biosynthetic pathway. PLoS One.

[41] Cheng A-X, Zhang X, Han X-J, Zhang Y-Y, Gao S, Liu C-J (2018). Identification of chalcone isomerase in the basal land plants reveals an ancient evolution of enzymatic cyclization activity for synthesis of flavonoids. New Phytol.

[42] Han Y, Vimolmangkang S, Soria-Guerra RE, Rosales-Mendoza S, Zheng D, Lygin AV (2010). Ectopic expression of apple F3’H genes contributes to anthocyanin accumulation in the arabidopsis tt7 mutant grown under nitrogen stress. Plant Physiol.

[43] Wang H, Fan W, Li H, Yang J, Huang J, Zhang P (2013). Functional characterization of dihydroflavonol-4-reductase in anthocyanin biosynthesis of purple sweet potato underlies the direct evidence of anthocyanins function against abiotic stresses. PLoS One.

[44] Wilmouth RC, Turnbull JJ, Welford RWD, Clifton IJ, Prescott AG, Schofield CJ (2002). Structure and mechanism of anthocyanidin synthase from Arabidopsis thaliana. Structure.

[45] Wang Z, Meng D, Wang A, Li T, Jiang S, Cong P (2013). The methylation of the PcMYB10 promoter is associated with green-skinned sport in max red bartlett pear. Plant Physiol.

[46] Zhao ZC, Hu GB, Hu FC, Wang HC, Yang ZY, Lai B (2012). The UDP glucose: Flavonoid-3-O-glucosyltransferase (UFGT) gene regulates anthocyanin biosynthesis in litchi (Litchi chinesis Sonn.) during fruit coloration. Mol Biol Rep.

[47] Zhao J, Dixon RA (2010). The “ins” and “outs” of flavonoid transport. Trends Plant Sci.

[48] Broun P (2004). Transcription factors as tools for metabolic engineering in plants. Curr Opin Plant Biol.

[49] Albert NW, Davies KM, Lewis DH, Zhang H, Montefiori M, Brendolise C (2014). A conserved network of transcriptional activators and repressors regulates anthocyanin pigmentation in eudicots. Plant Cell.

[50] Yao G, Ming M, Allan AC, Gu C, Li L, Wu X (2017). Map-based cloning of the pear gene MYB114 identifies an interaction with other transcription factors to coordinately regulate fruit anthocyanin biosynthesis. Plant J.

[51] Xi W, Feng J, Liu Y, Zhang S, Zhao G (2019). The R2R3-MYB transcription factor PaMYB10 is involved in anthocyanin biosynthesis in apricots and determines red blushed skin. BMC Plant Biol.

[52] Espley RV, Hellens RP, Putterill J, Stevenson DE, Kutty-Amma S, Allan AC (2007). Red colouration in apple fruit is due to the activity of the MYB transcription factor, MdMYB10. Plant J.

[53] Meisel L, Fonseca B, González S, Baeza-Yates R, Cambiazo V, Campos R (2005). A rapid and efficient method for purifying high quality total RNA from peaches (Prunus persica) for functional genomics analyses. Biol Res.

[54] Grabherr MG, Haas BJ, Yassour M, Levin JZ, Thompson DA, Amit I (2011). Full-length transcriptome assembly from RNA-Seq data without a reference genome. Nat Biotechnol.

[55] Haas BJ, Papanicolaou A, Yassour M, Grabherr M, Blood PD, Bowden J (2013). De novo transcript sequence reconstruction from RNA-seq using the Trinity platform for reference generation and analysis. Nat Protoc.

[56] Mistry J, Chuguransky S, Williams L, Qureshi M, Salazar GA, Sonnhammer ELL (2021). Pfam: The protein families database in 2021. Nucleic Acids Res.

[57] Edgar RC (2010). Search and clustering orders of magnitude faster than BLAST. Bioinformatics.

[58] Simão FA, Waterhouse RM, Ioannidis P, Kriventseva EV, Zdobnov EM (2015). BUSCO: assessing genome assembly and annotation completeness with single-copy orthologs. Bioinformatics.

[59] Hart AJ, Ginzburg S, Xu M (Sam), Fisher CR, Rahmatpour N, Mitton JB, et al. EnTAP: Bringing faster and smarter functional annotation to non-model eukaryotic transcriptomes. Mol Ecol Resour. 2020;20:591–604. doi:10.1111/1755-0998.13106. 10.1111/1755-0998.13106 31628884

[60] Jensen LJ, Julien P, Kuhn M, von Mering C, Muller J, Doerks T (2008). eggNOG: Automated construction and annotation of orthologous groups of genes. Nucleic Acids Res.

[61] Ashburner M, Ball CA, Blake JA, Botstein D, Butler H, Cherry JM (2000). Gene ontology: tool for the unification of biology. Nat Genet.

[62] Finn RD, Bateman A, Clements J, Coggill P, Eberhardt RY, Eddy SR (2014). Pfam: the protein families database. Nucleic Acids Res.

[63] Wu S, Zhu Z, Fu L, Niu B, Li W (2011). WebMGA: a customizable web server for fast metagenomic sequence analysis. BMC Genomics.

[64] Jin J, Tian F, Yang DC, Meng YQ, Kong L, Luo J (2017). PlantTFDB 4.0: Toward a central hub for transcription factors and regulatory interactions in plants. Nucleic Acids Res.

[65] Huang DW, Sherman BT, Lempicki RA (2009). Bioinformatics enrichment tools: paths toward the comprehensive functional analysis of large gene lists. Nucleic Acids Res.

[66] Livak KJ, Schmittgen TD (2001). Analysis of relative gene expression data using real-time quantitative PCR and the 2-ΔΔCT method. Methods.

