# Supplementary material for: De novo assembly of a fruit transcriptome set identifies AmMYB10 as a key regulator of anthocyanin biosynthesis in Aronia melanocarpa
Source: BMC Plant Biol. 2022 Mar 25;22:143. doi: 10.1186/s12870-022-03518-8 (PMC8951710; doi:10.1186/s12870-022-03518-8)
Supplement: Supplementary file 2 — Additional file 2: Supplementary Figure S1. Transcriptome assembly length and quality. Supplementary Figure S2. Species distribution of BLAST top hits against the NCBI NR protein database with an E value cut-off of 1e-5. Supplementary Figure S3. Assignment of unigenes into different categories based on GO terms. Supplementary Figure S4. Clustering of candidate genes based on expression pattern correlated with fruit development. Supplementary Figure S5. Expression levels of structural and regulator genes involved with anthocyanin biosynthesis from the fruits of A. melanocarpa (n=6) at four developmental stages. Supplementary Figure S6. Transcription factors distribution in different gene families. Counts of transcription factors within the 5,799 differentially expressed genes were identified by searching the top A. thaliana BLASTx hits for TAIR codes within the transcription factor database (Plant TFDB v3.0). [file 12870_2022_3518_MOESM2_ESM.pdf]

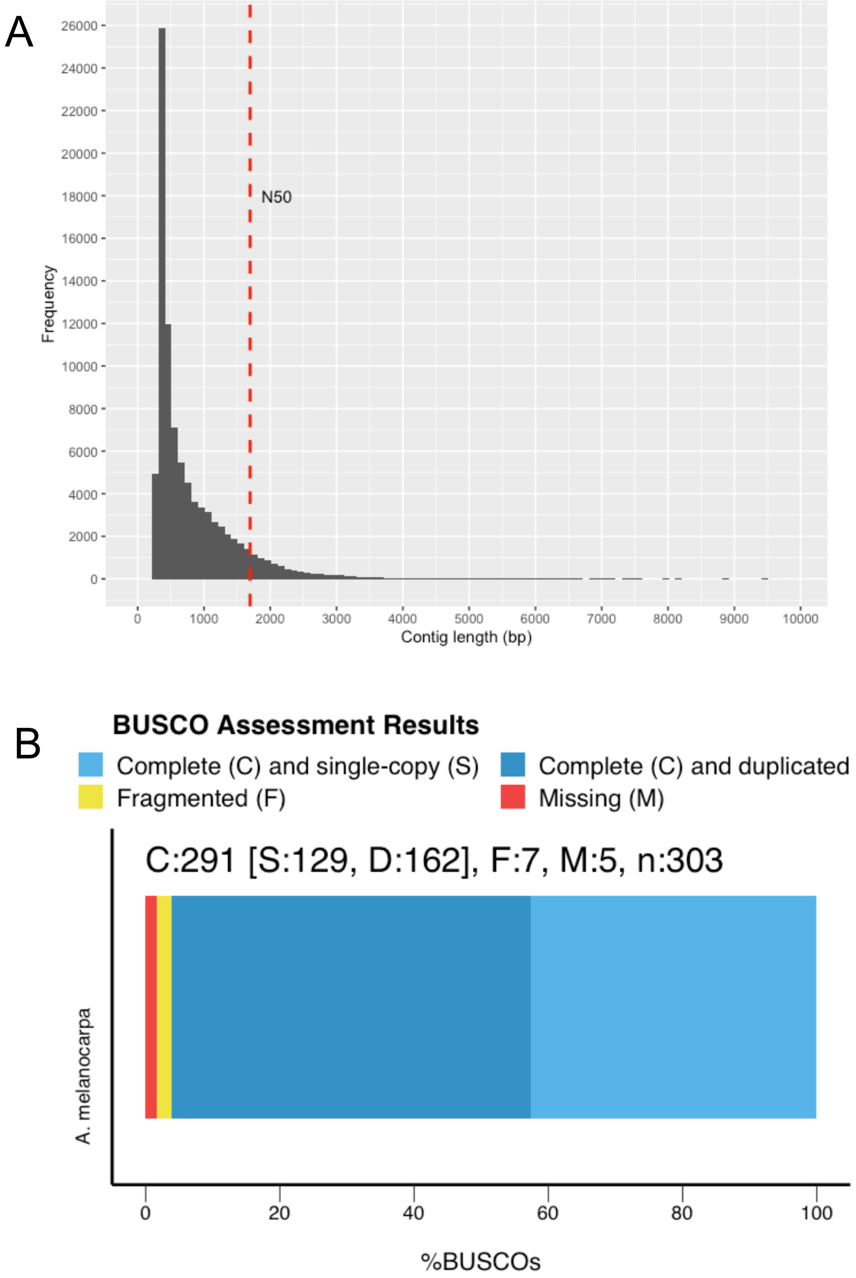

Supplementary Figure S1. Transcriptome assembly length and uality. (A) Contig length distribution with an 50 of , 95 bp is designated with a dotted vertical line. (B) Complete, fragmented, and missing Benchmar ing niversal Single Copy rthologs (B SC s) from the assembled A. melanocarpa de novo transcriptome.

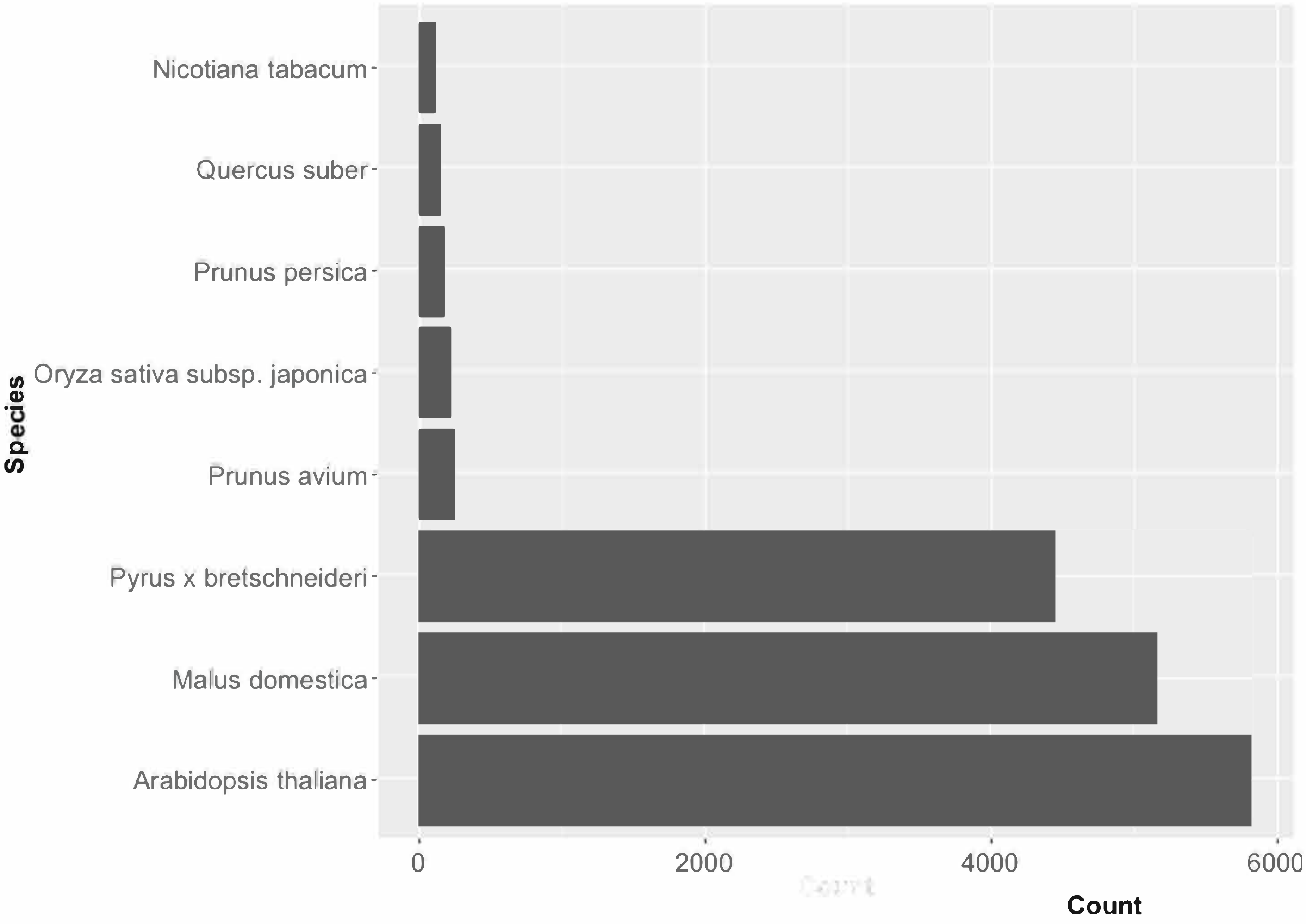

Supplementary Figure S2. Species distribution of BLAST top hits against the CBI R protein database with an value cut-off of e-5.

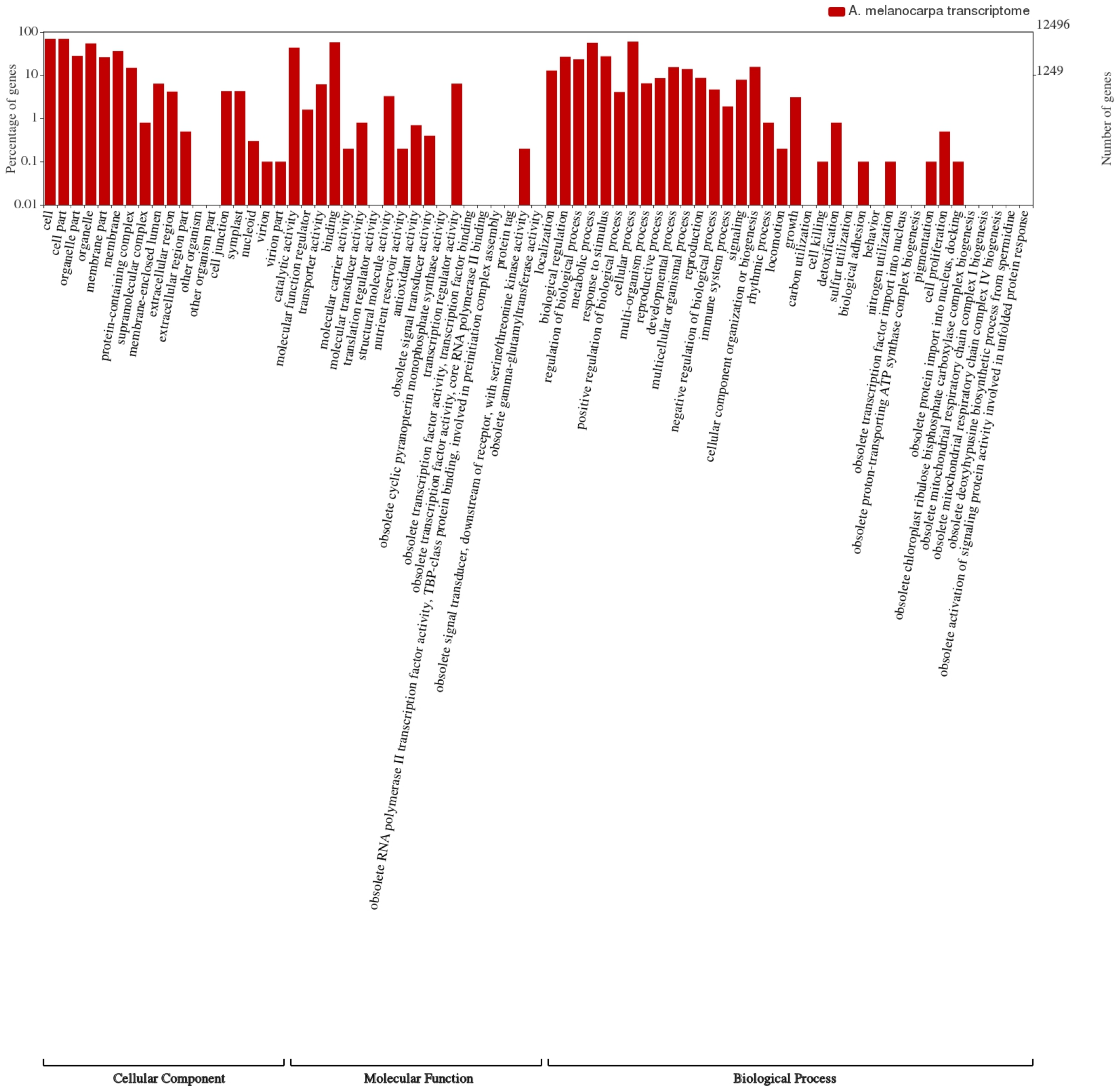

Supplementary Figure S3.
Assignment of unigenes into different categories based on terms.

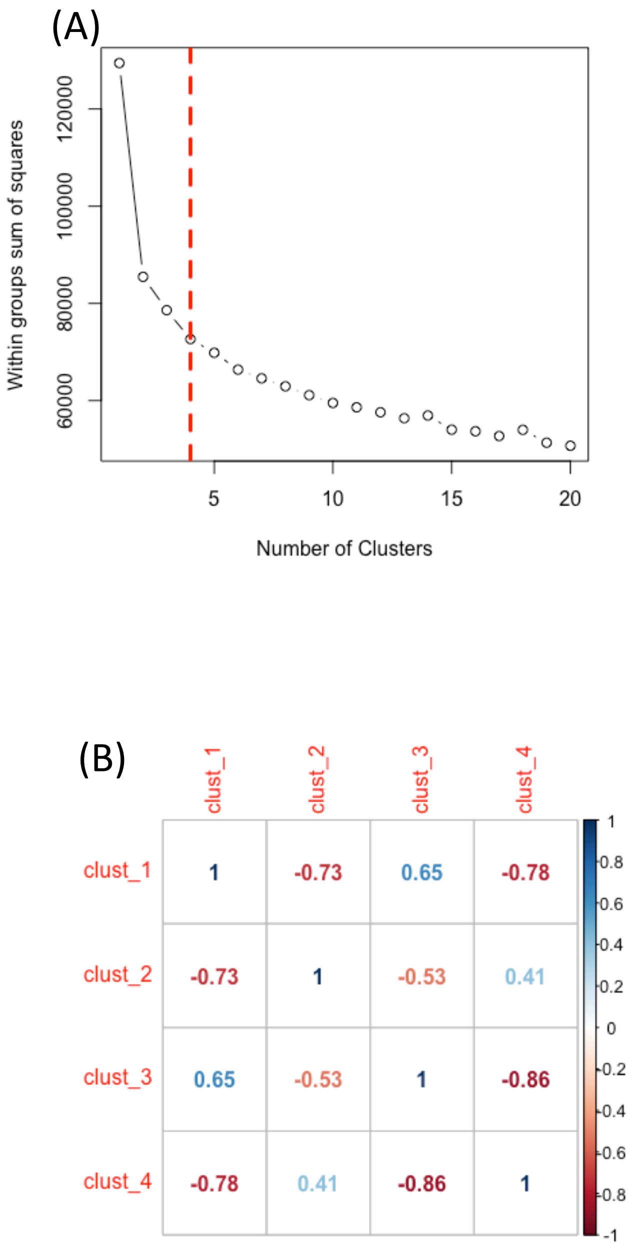

Supplementary Figure S4. Clustering of candidate genes based on expression pattern correlated with fruit development.

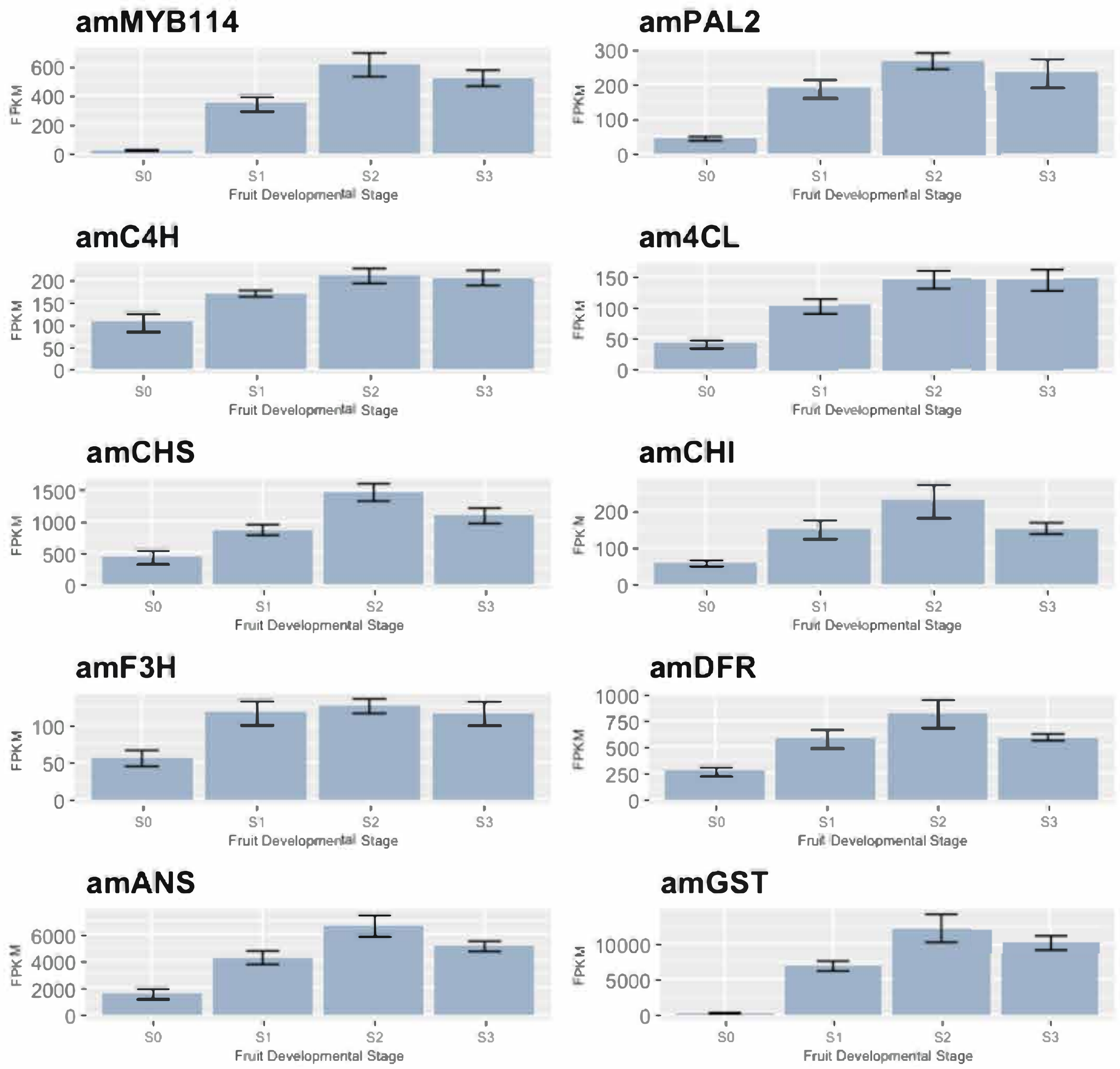

Supplementary Figure S5. Expression levels of structural and regulator genes involved with anthocyanin biosynthesis from the fruits of A. melanocarpa (n=6) at four developmental stages.

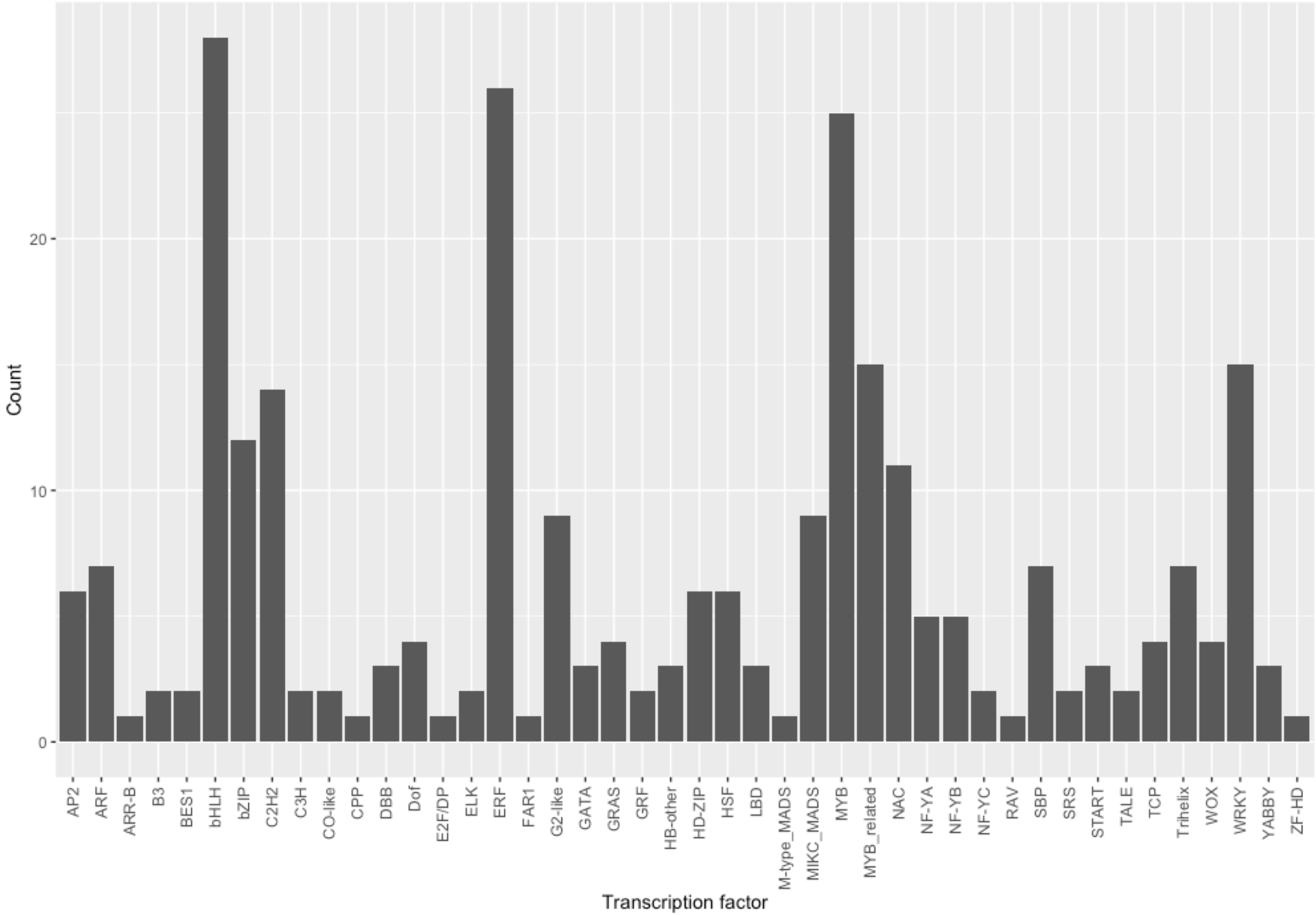

Supplementary Figure S6. Transcription factors distribution in different gene families. Counts of transcription factors within the 5,799 differentially expressed genes were identified by searching the top *A. thaliana* BLASTx hits for TAIR codes within the transcription factor database (Plant TFDB v3.0).
